# Short mindfulness meditation training: does it really reduce perceived stress?

**DOI:** 10.1007/s10339-022-01108-y

**Published:** 2022-09-21

**Authors:** Barbara Horrillo Álvarez, Carolina Marín Martín, Manuel Rodríguez Abuín, Laura Orio Ortiz

**Affiliations:** 1 Department of Personality, Assessment and Clinical Psychology, Faculty of Psychology, CES Cardenal Cisneros, Complutense University of Madrid, Madrid, Spain; 2 Department of Personality, Assessment and Clinical Psychology, Faculty of Psychology, Complutense University of Madrid, Madrid, Spain; 3 Teacher Training Center, Faculty of Education, Complutense University of Madrid, Madrid, Spain; 4 Department of Psychobiology and Methodology in Behavioral Science, Faculty of Psychology, Complutense University of Madrid, Madrid, Spain

**Keywords:** Attention, Attentive awareness, Stress reduction, Mindfulness meditation

## Abstract

To study whether an 8-week mindfulness meditation training program truly reduces perceived stress without designing a stress reduction program. An experimental study was performed in which we studied the effects of 8 weeks of MM training on attention and awareness, as measured by the MAAS (mindfulness attention awareness scale) and perceived stress, as measured by the PSQ (perceived stress questionnaire), in 80 volunteers from the general public recruited by email from university centers. An increase in the individual’s dispositional capacity to be attentive and aware of the experience of the present moment in everyday life was observed in the experimental group versus the control group; *F* (2, 156) = 14.30, *p* = .000, *η*2 partial = .155. Perceived stress showed no significant differences between groups in: social acceptance; *F* (2, 156) = 2.30, *p* = .103, overload; *F* (2, 156) = 2.32, *p* = .101, irritability, tension and fatigue; *F* (2, 156) = 2.27, *p* = .106, energy and joy; *F* (2, 156) = 2.79, *p* = .065. MM practice for 8 weeks of training increases the individual’s dispositional capacity to be attentive and aware of the experience of the present moment in everyday life but may not reduce perceived stress.

## Introduction

Mindfulness meditation (MM) comprises a series of practices aimed at developing mindfulness and awareness of the present moment in a non-analytical way, while avoiding ruminative thoughts (Saphiro 1982), without judgment (Brown and Ryan 2003), allowing self-observation and acceptance without trying to modify anything (Bishop et al. 2004). It could be considered a mind–body awareness that provides an opportunity to acquire and develop self-knowledge (Kabat-Zinn 2015). This *full attention* is the awareness that arises when attention is paid to the experience in the present moment, without judging or reacting to it (Kabat-Zinn 2015) with acceptance, which could imply the experiential non-avoidance that involves trying to alter the form, frequency or intensity of sensations, feelings or thoughts (Hayes et al. 2005). According to Vipassana theory, experiential avoidance exacerbates subjective stress, depression and anxiety (Bird et al. 2013), while in MM, the practitioner actively works with states of mind to remain peaceful with what occurs (Siegel et al. 2011).

At the end of the 1970s, MM expanded throughout the USA and Europe, pioneered by Kabat-Zinn, who introduced it into Western psychotherapy by including aspects of it in the educational program that he designed; mindfulness-based stress reduction (MBSR) (Kabat-Zinn 1984). Its efficacy has been proven as a protocol to reduce stress and increase well-being after 8 weeks of training (Shapiro et al. 2008).

The beneficial effects of MM practice have been well documented (Kabat-Zinn 2013), which include the ability of the individual’s dispositional capacity to be attentive and aware of the experience of the present moment in everyday life, a variable that may mediate or facilitate this effect (Grossman et al. 2004). The practice of MM produces an increase in the ability to remain attentive and aware in the present moment (Quaglia et al. 2016). This attention and full awareness would act to foster emotional regulation (Grecucci et al. 2015). Facilitating awareness or mindfulness is associated with moment-by-moment self-observation and acceptance of thoughts and emotions.

The practice of MM is also related to an increase in well-being (Brown and Ryan 2003), with more detailed perception, a decrease in negative effects, an increase in vitality and coping mechanisms (Grossman et al. 2004) and a decrease in stress-related discomfort (Martín-Asuero and García de la Banda 2007). Attention and awareness in the present moment is a capacity that can be developed (dispositional mindfulness) and could be associated with a response to physiological and adaptive stress (Kadziolka et al. 2016).

Meditative practice could influence the awareness that the subject has about himself and the way in which an experience undergone is processed, and this awareness could be key to its effectiveness in interventions related to stress reduction (Kinser et al. 2016).

## Mechanisms of the role of mindfulness in stress reduction

There is increasing scientific evidence of the relationship between stress (when it is maintained over time) and disease, since it produces organic, physiological, psychological and/or behavioral reactions that are harmful to health, so it is necessary to have resources to reduce this (Sarason and Sarason 2006). It is well known that repeated, excessive or prolonged stress reactivity could increase health risks (Cohen et al. 2017).

Different regulatory mechanisms have been proposed to explain how individuals can cope with stress. Some proposals from the cognitive approach emphasize coping based on a positive expectancy when facing situations of stress (e.g., Pulopulos et al. 2020). On the contrary, the proposals coming from meditation as stress regulators are not based on mechanisms based on a positive expectancy when facing emotional situations, but on the focus of the process beyond expectancy. As can be deduced from the systematic review by Azevedo et al. (2015), the mechanisms based on meditation techniques seem to indirectly affect the ability to buffer stress. In this case, it is not so much a change in the perception of expectations, but an activation and integration of the neural systems that influence attention, memory and emotional regulation. Stress regulation would be produced by an increase or activation of the neural mechanisms associated with these processes, through neuroplastic effects by activation of associated brain areas and circuits.

Similarly, as shown by Azevedo et al. (2015), when the mechanisms and systems involved in mindfulness meditation are distinguished from other meditation systems, there are some relevant findings. Unlike other active meditation techniques, in mindfulness meditation there is a specific activation in brain areas involved in sensory and emotional integration, as well as in self-control, body awareness and movement, thanks to the activity of the mid-cingulate cortex (MCC), the angular gyrus (AG), the primary and secondary sensorimotor cortex (SSI and II) and the premotor area (PMA).

Some meta-analyses indicate that meditative practice is one of the most widely used ways to alleviate the sustained effects of stress (Grossman et al. 2004). Over the course of recent decades, a host of MM programs have been designed to be applied as specific coadjuvant treatments for certain ailments, thus acquiring increasing theoretical and applied interest (Brown et al. 2007), and are now corelated to reducing stress, brain plasticity and gene expression (Creswell et al. 2012; Giuliani et al. 2011; Kaliman et al. 2014; Larouche et al. 2015).

Shapiro (1994) pointed out that meditative practice involves greater self-awareness and a reduction in defenses, which brings with it the emergence of latent personal problems. Thus, it is possible that people who practice it might experience stress or are overwhelmed by what emerges during the practice. This might appear contradictory, but a distinction should be made between protocols that include the practice of MM and practicing MM without including it in said protocols. One of the weaknesses in relation to the studies carried out on the practice of MM is that several 8-week training protocols in MM show effects on the reduction of perceived stress of beginner participants in MM practice (Baer et al. 2012). These results lead us to think that practicing MM for 8 weeks may reduce stress. However, these programs include other practices in addition to MM, which differ depending on the protocol applied and the intended effects, making it difficult to isolate the effects of MM.

## Variability in the protocols that include the practice of MM

One of the difficulties when comparing studies on the perceived effects on stress, as Horrillo et al. (2019) points out, may be that the intervention protocols that include MM during 8 weeks of training differ in some aspects; (a) the daily practice time assigned to participants, (b) the time assigned to weekly group sessions, (c) the type of practice that can be guided through audio recordings or in silence, (d) the inclusion of other practices in addition to MM itself, (e) the incorporation or not of MM withdrawal and (f) the amount to be paid for receiving MM training.

There is also variability in factors inherent to the participant, such as the actual daily practice time undertaken and that may or may not coincide with the time assigned, the motivation for the practice, the possible placebo effect, personality characteristics, etc. Other types of differences in the protocols are those stemming from the type of practice itself; (a) focus of attention (on an external or internal point) with eyes open or closed and (b) open monitoring, the practice with closed eyes that can also be static (Samatha) and dynamic (Kirtam Kriya), equally open-eyed practice can be static (Zen Koan) or dynamic (Tai Chi).

All this variability in the application of MM protocols entails a challenge for MM research (Rajaraman 2013), making the comparison between studies and the study of isolated MM effects difficult, since it is necessary to establish whether the effects of protocols that include MM training are due to the practice of MM itself or if there are other variables, such as those described above, which might alter and contaminate these effects. Scientific literature points out that physical and psychological collapses could be foreseeable if we could assess how stressful an experience or accumulation of experiences undergone are for a person (Sarason and Sarason 2006). Therefore, it is necessary to have mechanisms that help reduce stress when it is maintained over time.

## Aims of the research

This study aims to study whether the applied 8-week training program in MM produces effects on the ability of the individual’s dispositional capacity to be attentive and aware of the experience of the present moment in everyday life and on perceived stress. With the high number of variables described that operate in the different programs that include MM and that interact among themselves, it is difficult to distinguish which effects on stress are exclusively due to the practice of MM. Our goal was to measure the effects on stress without designing a stress reduction program (thus trying to reduce any possible placebo effect). Meditation aimed at reducing stress could generate confusion in the learner, given that the practice of MM on an 8-week training program will not necessarily produce this effect (Martín-Asuero and García-Banda 2010; Van Dam et al. 2014), although it might. MM should be practiced without a specific goal (Kabat-Zinn 2003), and the effects and possible benefits should arise as a consequence of continued practice in the discipline, not because of a possible suggestion relating the practice to relaxation and stress reduction.

The working hypothesis was that an increase in the ability of the individual’s dispositional capacity to be attentive and aware of the experience of the present moment in everyday life and aware now and a decrease in perceived stress would be observed in the experimental group compared with the control group on the wait list, after 8 weeks of training in MM.

## Method

### Participants

A total of 127 volunteers were recruited to participate in the research and were randomly assigned to the experimental group (*N* = 88) that practiced MM for 8 weeks, and to the wait list control group (*N* = 39) that practiced MM for 8 weeks after the experimental group finished their participation. There was an approximate ratio of 2:1 in the experimental group in comparison with the control group, as the dropout rate was expected to be higher in the experimental group than in the control group (e.g., Van Dam et al. 2014).

The only subjects considered for inclusion were those from the general public who had not previously practiced MM or any other form of meditative practice, so all participants were considered beginners. In addition, participants that either suffered from any type of mental disorder or consume anxiolytics and/or antidepressants were excluded. Eighty participants in the experimental group met both the inclusion and exclusion criteria. Of these 80 participants in the experimental group, 33 participants (41.25%) dropped out of training. 90.8% after the first and second week of training. Accordingly, the experimental group concluded with 47 participants.

The sample (*N* = 80) consisted of participants aged between 18 and 60. The experimental group (*N* = 47) comprised 40 women and 7 men (age; *M* = 26.30 and SD = 10.94) and the wait list control group (*N* = 33) included 28 women and 5 men (age; *M* = 23.52 and SD = 6.49).

Participants aged between 18 and 32 years old accounted for 88.7% of the sample, with the remaining 11.3% aged between 43 and 60 years old. A total of 75% of the sample were psychology students.

The socio-demographic characteristics of the sample are shown in Table 1. Student T and Cramer’s V tests were performed to study whether there were any differences between the experimental group and the wait list control group in terms of socio-demographic variables. No statistically significant differences were detected in either of them (*p* > 0.05).

**Table 1:** Sample Socio-demographic Characterization (*N* = 80)

Variables	Sample (*N* = 80)	Experimental group (*N* = 47)	Wait list control group (*N* = 33)	Statistics
*Age*				
*M*	25.15	26.30	23.52	*t* (76.31) = 1.396
SD	9.41	10.94	6.49	
Range	18–60	18–60	19–47	
*Gender*				
Female	68 (85%)	40 (85.1%)	28 (88.8%)	Cramer's *V* = .004
Male	12 (15%)	7 (14.9%)	5 (15.2%)	
*Marital status*				
Single	72 (90%)	41 (87.2%)	31 (93.9%)	Cramer's *V* = .125
Married	7 (8.8%)	5 (10.6%)	2 (6.1%)	
Divorced	1 (1.2%)	1 (2.1%)	0	
*Level of education*				
Secondary School	51 (63.8%)	29 (61.7%)	22 (66.7%)	Cramer's *V* = .246
3rd level graduate with Degree	22 (27.5%)	14 (29.8%)	8 (24.2%)	
Master’s Degree/postgraduate	6 (7.5%)	3 (6.4%)	3 (9.1%)	
PhD	1 (1.2%)	1 (2.1%)	0	
*Profession*				
Student	63 (78.7%)	37 (78.8%)	26 (78.8%)	Cramer's *V* = .256
Employed	10 (12.5%)	5 (10.6%)	5 (15.2%)	
Unemployed	7 (8.8%)	5 (10.6%)	2 (6.1%)	

### Procedure

The experimental study was approved by the Ethics Committee of the Faculty of Psychology at the Complutense University of Madrid. Subjects were recruited by means of an email inviting them to participate in the research, sent by the Faculty of Psychology at the Complutense University of Madrid to faculty staff, psychology and speech therapy students and their acquaintances, and by the Cardenal Cisneros Higher Education Centre to its psychology students.

Those interested in participating were informed about the study, given an appointment to start the research and randomly assigned to the experimental or wait list control groups. All participants signed an information sheet and informed consent form and completed an ad hoc questionnaire designed to collect their socio-demographic data and determine whether or not they met the study inclusion criteria. These criteria were: being in good health, of legal age and interested in receiving MM training for 8 weeks. Exclusion criteria were: having previously practiced MM, yoga, Tai Chi or another meditation-related practice, taking psychotropic drugs such as anxiolytics or antidepressants, or suffering from any type of mental disorder.

They then completed the research questionnaires before the start of training (pretest measure), at 4 weeks (intermediate measure) and at 8 weeks (posttest measure).

The MM training consisted of a 1-h group session per week for 8 weeks. In this session, MM was taught and practiced, any questions or difficulties were resolved and the weekly practice was discussed in terms of experiences during the week and the practice carried out in the weekly meeting. Participants were instructed to practice daily for as long as possible without exceeding 30 min. This maximum time limit was established as a precaution against the possible adverse effects of meditation on people with psychopathology or a predisposition to it and to operationalize the time criterion for practice. Practice time was assessed through a self-recording system in which each participant included the exact start and end time for each day of practice throughout the 8 weeks of training. The training was delivered by a member of the research team who had experience in MM practice at a personal and professional level.

The MM applied in this research focused attention on diaphragmatic breathing (Almendro and López 2016; Moñivas et al. 2012; López 2016), the origin of which is Zen meditation (Austin 1999). The design of the training program was based on the one applied in the psychotherapeutic context of the Oxígeme Process (Almendro 2012; Almendro and López 2016). This protocol includes the practice of MM as applied in MBSR programs (Kabat-Zinn 1984), but differs from these in that it does not include other therapeutic practices (body scan, physical relaxation, stress reduction talks, etc.), practice is not focused on the specific goal of stress reduction, and meditation is performed in silence.

### Instruments

#### Mindfulness attention awareness scale (MAAS)

This consists of 15 items in its Spanish version (Soler et al. 2012), adapted from the original version by Brown and Ryan (2003). The scale assesses the individual’s dispositional capacity to be attentive and aware of the experience of the present moment in everyday life. Its internal consistency (Cronbach’s *α*) is 0.89 (0.88 for this sample).

#### *Perceived stress questionnaire* (PSQ)

This consists of 30 items in its Spanish version (Sanz-Carrillo et al. 2002), adapted from the original version by Levenstein et al. (1993). The questionnaire is used to measure stress in clinical psychosomatic research. It contains the following 6 subscales: social acceptance, overload, irritability, tension and fatigue, energy, joy, fear and anxiety, and self-realization and satisfaction. Internal consistency (Cronbach’s *α*) is 0.90 for the original version and 0.87 for the version adapted to Spanish (0.93 for this sample). Cronbach’s α in this sample was: social acceptance (0.77), overload (0.77), irritability, tension and fatigue (0.85), energy, joy (0.77), fear and anxiety (0.45), and self-realization and satisfaction (0.60). The latter two were eliminated due to their low reliability (Peterson, 1994).

### Data analysis

To perform the statistical analysis of the ability of the individual’s dispositional capacity to be attentive and aware of the experience of the present moment in everyday life and perceived stress, an experimental design was used applying a mixed ANOVA with one independent measurement factor (experimental group and wait list control) and one repeated measurement factor (the three time points). The interaction effect refers to the passage of time over the 8 weeks of training by the group they belong to (time*group). The main effect of the measurements refers to the measurements taken at the three time points during the 8 weeks of training. The main group effect refers to having practiced MM or not, that is, to belonging to the experimental and wait list control groups. Mauchly’s W indicated that the assumption of sphericity was met in all cases.

The *post hoc* comparisons were made taking into account the Bonferroni adjustment. In all statistical tests, a confidence level of 95% was applied, (*α* = 0.05). The SPSS Statistics version 24 program was used.

## Results

### MAAS results

Descriptive statistics are detailed in Table 2.

**Table 2:** Descriptive statistics on the individual’s dispositional capacity to be attentive and aware of the experience of the present moment in everyday life (MAAS) and the subscales (PSQ); social acceptance, overload, irritability, tension and fatigue and energy and joy in the experimental group and the wait list group during 8 weeks of training in mindfulness meditation

Variable	Experimental group (*N* = 47)		Wait list control group (*N* = 33)		Sample (*N* = 80)	
	*M*	SD	*M*	SD	*M*	SD
*MAAS*						
Attention and awareness pretest	51.25	13.62	53.51	13.44	52.18	13.50
Attention and awareness intermediate	57.12	12.64	54.48	14.08	56.03	13.23
Attention and awareness posttest	61.04	12.72	50.48	15.25	56.68	14.69
*PSQ*						
Social acceptance pretest	13.46	4.11	13.72	3.81	13.57	3.96
Social acceptance intermediate	12.14	2.91	13.30	3.83	12.62	3.35
Social acceptance posttest	12.00	3.80	13.78	4.24	12.73	4.06
Overload pretest	9.78	2.93	10.93	2.27	10.26	2.72
Overload intermediate	9.14	2.64	9.18	2.33	9.16	2.50
Overload posttest	8.82	2.53	9.66	2.64	9.17	2.59
Irritability, tension and fatigue pretest	23.97	6.26	26.30	5.24	24.93	5.93
Irritability, tension and fatigue intermediate	21.46	5.81	24.84	6.42	22.86	6.26
Irritability, tension and fatigue posttest	20.44	4.71	25.27	6.41	22.43	5.94
Energy and joy pretest	12.34	2.86	13.15	3.05	12.67	2.95
Energy and joy intermediate	11.68	3.00	12.75	2.98	12.12	3.02
Energy and joy posttest	10.87	2.81	12.96	3.05	11.73	3.08

No statistically significant group main effect was found, *F* (1, 78) = 1.77, *p* = 0.187. A statistically significant main effect of the measurements of the 8 weeks of training was found, *F* (2, 156) = 5.27, *p* = 0.006, partial *η*2 = 0.063, with a higher median posttest. A statistically significant interaction effect was found between the 8 weeks of training and group, *F* (2, 156) = 14.30, *p* < 0.001, *η*2 partial = 0.155. The *post hoc* comparisons of the interaction effect revealed statistically significant differences in the experimental group between the pretest and intermediate, *p* = 0.001, between the intermediate and posttest, *p* = 0.025 and between the pretest and posttest, *p* < 0.001.

### PSQ results

#### Social acceptance subscale

Descriptive statistics are detailed in Table 2.

No statistically significant group main effect was found, *F* (1, 78) = 2.00, *p* = 0.160. A statistically significant main effect of the measurements was found in the 8 weeks of training, *F* = (2, 156) = 3.34, *p* = 0.038, partial *η*2 = 0.041, with a higher median posttest. No statistically significant interaction effect was found between the 8 weeks of training and the group, *F* (2, 156) = 2.30, *p* = 0.103. The *post hoc* comparisons of the main effect of measurements indicated statistically significant differences between the pretest and intermediate, *p* = 0.021.

### Overload subscale

Descriptive statistics are detailed in Table 2. No statistically significant group main effect was found, *F* (1, 78) = 1.80, *p* = 0.183. A statistically significant main effect of the measurements was found in the 8 weeks of training, *F* (2, 156) = 12.51, *p* < 0.001, partial *η*2 = 0.14, with a higher median pretest. No statistically significant interaction effect was found, *F* (2, 156) = 2.32, *p* = 0.101. The *post hoc* comparisons of the main effect of measurements indicated statistically significant differences between the pretest and intermediate, *p* < 0.001, between the pretest and posttest, *p* < 0.001 and between the intermediate and posttest, *p* < 0.001.

### Irritability, tension and fatigue subscale

Descriptive statistics are detailed in Table 2.

A statistically significant group main effect was found, *F* (1, 78) = 9.62, *p* = 0.003, partial *η*2 = 0.11, with a higher median in the wait list control group. A statistically significant main effect of the measurements of the 8 weeks of training was found, *F* = (2, 156) = 8.88, *p* < 0.001, partial *η*2 = 0.10, with a higher median pretest. The *post hoc* comparisons of the group main effect revealed statistically significant differences between the experimental group and the wait list control group, *p* = 0.003. No statistically significant interaction effect was found, *F* (2, 156) = 2.27, *p* = 0.106.

### Energy and joy subscale

Descriptive statistics are detailed in Table 2. A statistically significant group main effect was found, *F* (1, 78) = 5.19, *p* = 0.025, partial *η*2 = 0.062, with a higher median in the wait list control group. A statistically significant main effect of the measurements was found in the 8 weeks of training, *F* = (2, 156) = 4.22, *p* = 0.016, partial *η*2 = 0.05, with a higher median pretest. No statistically significant interaction effect was found, *F* (2, 156) = 2.79, *p* = 0.065. The *post hoc* comparisons of the main effect of measurements indicated statistically significant differences between the pretest and posttest moments, *p* = 0.018. The *post hoc* comparisons of the group main effect revealed statistically significant differences between the experimental group and the wait list control group, *p* = 0.025.

A statistically significant interaction effect was thus only observed in the variable of attention and awareness in the present moment *p* < 0.001 (measured via MAAS).

## Discussion

In this study, we tested the effects of 8 weeks of MM training on the ability of the individual’s dispositional capacity to be attentive and aware of the experience of the present moment in everyday life and on perceived stress.

After 8 weeks of training, the experimental group showed an increased ability in the individual’s dispositional capacity to be attentive and aware of the experience of the present moment in everyday life compared with the wait list control group, suggesting that MM as applied in this study developed this practice-defining quality. These results are consistent with those obtained in other similar studies (Van Dam et al. 2014) and meta-analyses (Quaglia et al. 2016).

In relation to perceived stress, the results appear to indicate that there was no reduction in such stress, which is inconsistent with the results obtained in other studies (Baer et al. 2012; Bränström et al. 2010; Eberth and Sedlmeier 2012; Galantino et al. 2005; Greeson and Brantley 2011; Grossman et al. 2004; Keng et al. 2011; Nyklícek and Kuijpers 2008). However, recent research was based on programs aimed at reducing stress, suggesting that stress reduction may not be linked to the practice of MM but to the other practices used or to a combination of these. In contrast, the results were consistent with those obtained by other authors (Grecucci 2015; Kabat-Zinn 2003; Van Dam et al. 2014). It is possible that perceived stress may have a positive relationship with mindfulness, even 18 months after an MBCT training program (De Zoysa et al. 2014).

These findings may be partially explained by the fact that MM is not a stress-reducing practice per se, although it may generate this effect. It should be noted that the protocol applied here was not focused on reducing stress (although its effects were measured), like other protocols designed for this purpose. Practicing MM with the aim of reducing stress could cause confusion in beginners, since the practice of MM in a training of only 8 weeks is not designed to produce these effects (Van Dam et al. 2014).

The non-decrease in perceived stress in this study can also be explained by the more complex regulatory and integrative psychological processes that occur in MM. As shown in the work of Pulopulos et al. (2020), changes in the expectation of being able to deal with stress can generate differences in salivary cortisol, as well as in anticipatory cognitive coping, in some aspects of psychological stress and in heart rate variability. That is, a negative or positive bogus feedback of the ability to deal with stressful events can produce physiological, psychological and biochemical changes related to coping with stress. However, when it comes to MM, there is not this short-term effect generated by a specific change in an expectancy of being able to deal with a difficult or stressful task; in MM the changes in stress coping seem to be derived from changes in the integration and coordination of sensory and emotional processing, as well as in self-control, body awareness and movement, as suggested by Azevedo et al. (2015). This integration of processes is what would allow a buffer effect of stress by the MM, something on the other hand very complex to do in an 8-week meditation program. Perhaps, the results that support stress reduction in 8 weeks in other MM programs have to do not only with other components involved, but also with the generation of a positive expectancy about the skills and benefits that the trainee believes will be achieved.

The heterogeneous results obtained in the questionnaire on the ability to remain attentive and aware (awareness) at the present moment in the participants could perhaps be explained because, in some of them, becoming aware implies thoughts, feelings, sensations and perceptions which induce greater levels of stress during the first few weeks of practice than at the start of the practice, although, subsequently, with more continuous practice over time, this will be reduced.

It is worth noting that only 20% of studies on the beneficial effects of MM included a wait list control group and only 9% an active control group (Van Dam et al. 2018). A standard control group may have no interest in MM, and therefore, research study groups may be more heterogeneous. Future research should continue to use a wait list control group but add an active control group.

Regular practice of MM may enhance the individual’s dispositional capacity to be attentive and aware of the experience of the present moment in everyday life, which could facilitate greater self-knowledge and reduce the tendency to avoidance (Hervás et al. 2016), which in turn could be linked to a reduction in stress in the medium but not short term.

It should be noted that the best effects that MM entails occur when accompanied by psychotherapy (McGee 2008). The suggestion is to integrate the effects that are obtained with the practice of MM within a therapeutic context into psychotherapy, since practicing MM without therapeutic accompaniment in beginners could contribute to not achieving its benefits. It is possible that even if participants had practiced MM for longer in the 8 weeks, the results would not have changed, as it appears that the mindfulness skills from which the other effects of MM may be derived in novice practitioners may involve *top-down* emotional regulation mechanisms (associated with cognitive control), whereas experienced practitioners would be more likely to use *bottom-up* mechanisms (linked to the perceptual system) (Grecucci 2015).

In future research, it would be interesting to extend the study follow-up to assess the possible medium- and long-term effects of MM training and analyze whether the results obtained are maintained or diminish over time. It would also be useful to apply multivariate techniques, such as MANOVA, as these might yield interesting results, although given the characteristics of the sample (N and groups), the conclusions might be different. Another possibility for future research would be to investigate the effects of 8 weeks of MM training on these and other variables in clinical trials that eliminate any practice other than MM itself, so as not to alter the results and enable an accurate determination of its true effects, as the present study suggests that 8 weeks of MM training increases attention and awareness in the present moment, but does not reduce stress in beginners.

Lastly, it should be noted that although more research is required on the effects of the 8-week MM training program, it does not include other practices. It seems that this short period of time does not necessarily provide the beneficial effects on stress that have been attributed to it.

A key aspect to consider and that could be related to the results obtained is that the formal practice of MM (the act of meditating) produces effects when they extend outside the established practice time, that is, when MM is already incorporated into everyday life (Shapiro et al. 2007).

### Limitations

- The research uses self-reports as measurement of the variables. The inclusion of psycho-physiological tests is suggested as it would contribute to measure perceived stress such as heart rate, respiratory rate or electrodermal conductance to contrast and complement the results obtained.
- Although the sample size is moderate (*N* = 80), we propose replicating the study with a larger sample size to compare the results.
- A waiting list control group has been included, but for future research it would be advisable to also incorporate an active control group.
- To carry out the experimental study, participants were asked to practice for a maximum of 30 consecutive minutes daily. This time was determined taking into account that the participants were novices in the practice and that MM was practiced in silence and not by means of an audio guide. This maximum time was also assigned for operational reasons of the research. However, a minimum time was not set and, if incorporated in future research, it would contribute to standardize the daily practice length of the sample.
- Seventy-five percent of the sample were psychology students, which limits the variability in the sample profile and adds a possible variable due to previous theoretical knowledge they may have about MM practice. Therefore, for future research a recommendation would be to include more varied profiles.
